# Review of the Ticagrelor Trials Evidence Base

**DOI:** 10.1161/JAHA.123.031606

**Published:** 2024-05-28

**Authors:** Grace C. Herron, Eric R. Bates

**Affiliations:** ^1^ University of Michigan Medical School Ann Arbor MI USA; ^2^ Division of Cardiovascular Medicine, Department of Internal Medicine University of Michigan Ann Arbor MI USA

**Keywords:** clopidogrel, P2Y_12_ receptor inhibitor, prasugrel, ticagrelor, Coronary Artery Disease

## Abstract

Ticagrelor is a platelet P2Y_12_ receptor inhibitor approved for use in patients with acute coronary syndromes, coronary artery disease, and low‐moderate risk acute ischemic stroke or high‐risk transient ischemic attack. Clinical trials have evaluated the efficacy and safety of ticagrelor on ischemic and bleeding outcomes for different indications and with varying treatment approaches. As a result, there is a large body of clinical evidence demonstrating different degrees of net clinical benefit compared with other platelet inhibitor drugs based on indication, patient characteristics, clinical presentation, treatment duration, and other factors. We provide a review of the major trials of ticagrelor in the context of other randomized trials of clopidogrel and prasugrel to organize the volume of available information, elevate corroborating and conflicting data, and identify potential gaps as areas for further exploration of optimal antiplatelet treatment.

**Nonstandard Abbreviations and Acronyms**
BARC: Bleeding Academic Research Consortium
DAPT: dual antiplatelet therapy

Ticagrelor is an oral platelet P2Y_12_ receptor inhibitor. It is the first of a new chemical class of antiplatelet agents, the cyclopentyltriazolopyrimidines, and binds reversibly to the P2Y_12_ receptor to prevent ADP‐mediated platelet activation and inhibit platelet aggregation.^1^, ^2^ Clopidogrel and prasugrel are thienopyridine agents that also inhibit the P2Y_12_ receptor but do so irreversibly. They are also prodrugs requiring metabolic activation by hepatic CYP2C19, whereas ticagrelor is orally active. Clopidogrel activation is impaired in patients with CYP2C19 loss‐of‐function alleles, potentially limiting therapeutic efficacy. Compared with clopidogrel, ticagrelor has faster onset of action, greater potency, and less response variability. Therefore, ticagrelor has pharmacodynamic advantages as a P2Y_12_ inhibitor compared with clopidogrel when combined with aspirin as dual antiplatelet therapy (DAPT). However, when compared with clopidogrel and prasugrel in clinical trials, ticagrelor has produced efficacy and safety outcomes that vary by indication, patient characteristics, clinical presentation, treatment duration, and other factors. Moreover, ischemic risk has declined in the current treatment era and recent reports have suggested that a major bleeding event has at least the same or greater prognostic impact on mortality risk as myocardial infarction (MI), potentially decreasing the clinical benefit of a more potent antiplatelet agent.^3^, ^4^, ^5^ As a result, bleeding avoidance strategies and net clinical benefit have become increasingly important clinical considerations in determining antiplatelet therapy strategy.

This review provides a summary of the major ticagrelor randomized clinical trials with enrollment exceeding 400 participants (Figure 1) that have supported clinical use and guideline recommendations.^6^, ^7^, ^8^, ^9^, ^10^, ^11^ We excluded trials with pharmacodynamic or pharmacokinetic end points as a primary outcome. A total of 22 trials were identified that evaluated patients treated for acute coronary syndrome (ACS), stroke or transient ischemic attack (TIA), peripheral artery disease, coronary artery disease (CAD), secondary prevention, DAPT de‐escalation, or aspirin withdrawal therapy (Figure 2). The trial acronyms in this review are tabulated in Table 1. The ticagrelor clinical trial statistical results are summarized in Tables 2, 3, 4, 5, 6. Corresponding trials with clopidogrel or prasugrel are briefly summarized to provide clinical context.

**Figure 1:**
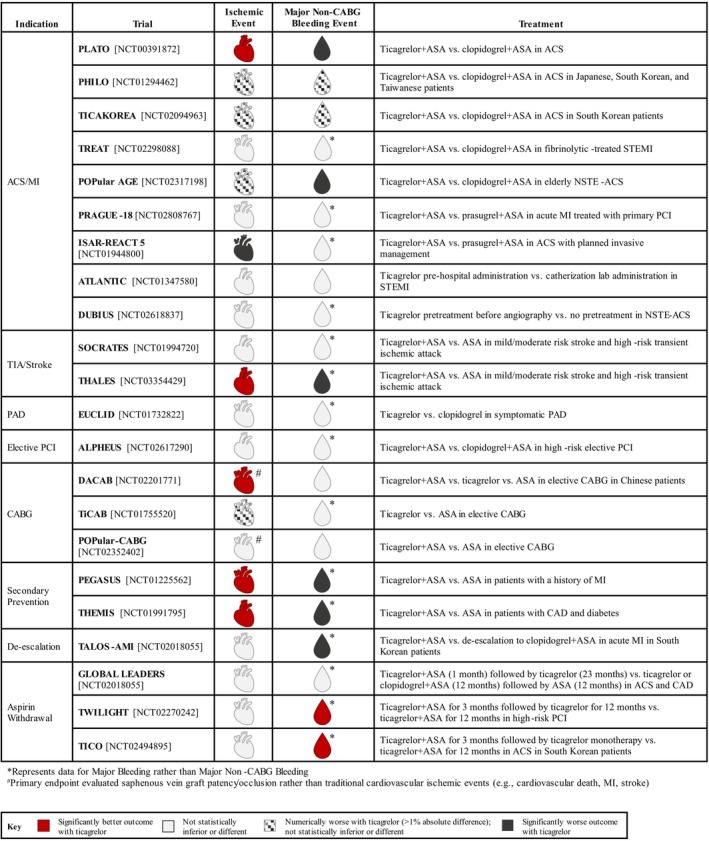
Selected ticagrelor randomized clinical trials. ACS indicates acute coronary syndrome; CABG, coronary artery bypass graft; CAD, coronary artery disease; MI, myocardial infarction; NSTE‐ACS, non‐ST elevation acute coronary syndrome; PAD, peripheral artery disease; PCI, percutaneous coronary intervention; and STEMI, ST‐segment elevation myocardial infarction.

**Figure 2:**
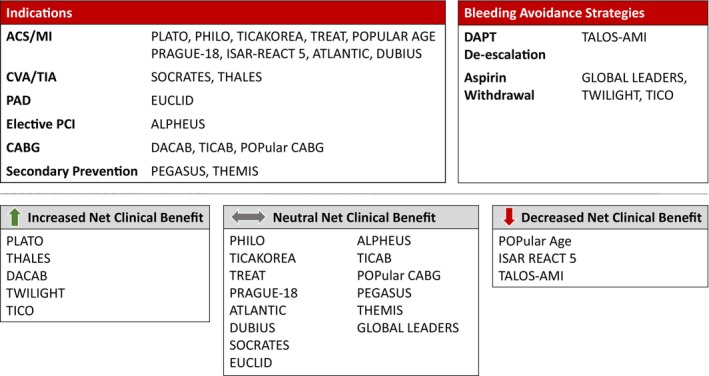
Net clinical benefit with ticagrelor in randomized clinical trials. ACS indicates acute coronary syndrome; CABG, coronary artery bypass graft; CVA, cerebral vascular accident; DAPT, dual antiplatelet therapy; MI, myocardial infarction; PAD, peripheral artery disease; PCI, percutaneous coronary intervention; MI, myocardial infarction; and TIA, transient ischemic attack.

**Table 1:** Selected P2Y_12_ Receptor Inhibitor Randomized Clinical Trial Acronyms

***Ticagrelor trials*** **ALPHEUS:** Assessment of Loading with the P2Y_12_ Inhibitor Ticagrelor or Clopidogrel to Halt Ischemic Events in Patients Undergoing Elective Coronary Stenting **ATLANTIC:** Administration of Ticagrelor in the Cath Lab or the Ambulance for New ST Elevation Myocardial Infarction to Open the Coronary Artery **DACAB:** Different Antiplatelet Therapy Strategy After Coronary Artery Bypass Graft Surgery **DUBIUS:** Downstream versus Upstream Strategy for the Administration of P2Y_12_ Receptor Blockers in Non‐ST Elevated Acute Coronary Syndromes with Initial Invasive Indication **EUCLID:** Examining Use of Ticagrelor in Peripheral Artery Disease **GLOBAL LEADERS:** A Clinical Study Comparing Two Forms of Antiplatelet Therapy After Stent Implantation **ISAR‐REACT 5:** Intracoronary Stenting and Antithrombotic Regimen: Rapid Early Action for Coronary Treatment **PEGASUS‐TIMI 54:** Prevention of Cardiovascular Events in Patients with Prior Heart Attack Using Ticagrelor Compared to Placebo on a Background of Aspirin–Thrombolysis in Myocardial Infarction 54 **PHILO:** Ticagrelor vs. Clopidogrel in Japanese, Korean and Taiwanese Patients with Acute Coronary Syndrome **PLATO:** Study of Platelet Inhibition and Patient Outcomes **POPular AGE:** Clopidogrel Versus Ticagrelor or Prasugrel in Patients Aged 70 y or Older with Non‐ST‐Elevation Acute Coronary Syndrome **POPular CABG:** The Effect of Ticagrelor on Saphenous Vein Graft Patency in Patients Undergoing Coronary Artery Bypass Grafting Surgery **PRAGUE‐18:** Comparison of Prasugrel and Ticagrelor in the Treatment of Acute Myocardial Infarction **SOCRATES:** Acute Stroke or Transient Ischaemic Attack Treated with Aspirin or Ticagrelor and Patient Outcomes **TALOS‐AMI:** Ticagrelor Versus Clopidogrel in Stabilized Patients with Acute Myocardial Infarction **THALES:** Acute Stroke or Transient Ischaemic Attack Treated with Ticagrelor and aspirin for Prevention of Stroke and Death **THEMIS:** The Effect of Ticagrelor on Health Outcomes in Diabetes Mellitus Patients Intervention Study **TICAB:** Study Comparing Ticagrelor with Aspirin for Prevention of Vascular Events in Patients Undergoing CABG **TICAKOREA:** Ticagrelor Versus Clopidogrel in Asian/Korean Patients with ACS Intended for Invasive Management **TICO:** Ticagrelor Monotherapy After 3 m in the Patients Treated with New Generation Sirolimus Stent for Acute Coronary Syndrome **TREAT:** Ticagrelor in Patients with ST‐Elevation Myocardial Infarction Treated with Pharmacological Thrombolysis **TWILIGHT:** Ticagrelor with Aspirin or Alone in High‐Risk Patients After Coronary Intervention ***Clopidogrel and Prasugrel Trials*** **CHANCE:** Clopidogrel in High‐Risk Patients with Acute Nondisabling Cerebrovascular Events **CHANCE‐2:** Ticagrelor or Clopidogrel with Aspirin in High‐Risk Patients with Acute Nondisabling Cerebrovascular Events II **CURE:** Clopidogrel in Unstable Angina to Prevent Recurrent Events **HOST‐EXAM:** Harmonizing Optimal Strategy for Treatment of Coronary Artery Stenosis—Extended Antiplatelet Monotherapy **HOST‐REDUCE‐POLYTECH‐ACS:** Harmonizing Optimal Strategy for Treatment of Coronary Artery Diseases‐Comparison of Reduction of Prasugrel Dose or Polymer Technology in ACS Patients **MASTER‐DAPT:** Management of High Bleeding Risk Patients Post Bioresorbable Polymer Coated Stent Implantation with an Abbreviated versus Standard DAPT Regimen **PHARMCLO:** Pharmacogenetics of Clopidogrel in Patients with Acute Coronary Syndromes **POINT:** Platelet‐Oriented Inhibition of New TIA and Minor Ischemic Stroke **POPular Genetics:** CYP2C19 Genotype‐Guided Antiplatelet Therapy in ST‐Segment Elevation Myocardial Infarction Patients—Patient Outcome after Primary PCI **SMART CHOICE:** Smart Angioplasty Research Team: Comparison Between P2Y12 Antagonist Monotherapy Versus Dual Antiplatelet Therapy in Patients Undergoing Implantation of Coronary Drug‐Eluting Stents **STOPDAPT‐2:** Short and Optimal Duration of Dual Antiplatelet Therapy After Everolimus‐Eluting Cobalt‐Chromium Stent **STOPDAPT‐2 ACS:** Short and Optimal Duration of Dual Antiplatelet Therapy for the Patients with ACS **TAILOR PCI:** Tailored Antiplatelet Initiation to Lessen Outcomes Due to Decreased Clopidogrel Response After Percutaneous Coronary Intervention **TRITON‐TIMI 38:** Trial to Assess Improvement in Therapeutic Outcomes by Optimizing Platelet Inhibition with Prasugrel—Thrombolysis in Myocardial Infarction 38 **TOPIC:** Timing of Platelet Inhibition after Acute Coronary Syndrome **TROPICAL‐ACS:** Testing Responsiveness to Platelet Inhibition on Chronic Antiplatelet Treatment for Acute Coronary Syndromes

## Acute Coronary Syndromes

DAPT with clopidogrel compared with aspirin monotherapy was initially shown to reduce the risk of major adverse cardiovascular events in patients after non–ST‐segment elevation ACS in the CURE trial,^12^ but with increased risk of major and minor bleeding. DAPT with prasugrel in the TRITON‐TIMI 38 trial^13^ and ticagrelor in the PLATO trial^14^ subsequently was shown to further reduce the risk of major adverse cardiovascular events, but with the risk of additional increased major and minor bleeding.

### Comparison of Ticagrelor With Clopidogrel

The US Food and Drug Administration first approved ticagrelor in July 2011 to reduce the risks of cardiovascular death and MI in patients with ACS based on results from the PLATO trial that compared DAPT with ticagrelor or clopidogrel (Table 2).^14^ The benefit of ticagrelor over clopidogrel in the composite primary outcome of cardiovascular death, MI, or stroke at 12 months was present with or without ST‐segment elevation and with or without percutaneous coronary intervention (PCI). Additionally, the risk of stent thrombosis was reduced and there was a surprising reduction in cardiovascular death, a finding that has not been reproduced in any other DAPT trial. Further analysis showed no difference in stroke rates. Maintenance doses of aspirin above 100 mg were associated with decreased ticagrelor effectiveness in a prespecified subgroup analysis,^15^ one potential explanation for the lack of benefit in patients enrolled in North America where aspirin doses were high, but the biological significance of the putative interaction remains unexplained, so chance alone cannot be excluded. There was no increased risk of PLATO major bleeding with ticagrelor, but there were increased rates of major and minor bleeding in patients not undergoing coronary artery bypass graft surgery (CABG) and in dyspnea.

**Table 2:** Acute Coronary Syndrome RCTs

Trial Acronym	PLATO (14)	PHILO (17)	TICAKOREA (18)	TREAT (19)	POPular AGE (21)
Total patients	18 624	801	800	3799	1002
Study design	Double‐blind	Double‐blind	Open‐label	Open‐label	Open‐label
Enrollment period	October 2006–July 2008	February 2011–July 2012	July 2014–June 2017	November 2015–November 2017	June 2013–October 2018
Publication year	2009	2015	2019	2018	2020
Location	Multinational	Japan, Taiwan, South Korea	South Korea	Multinational	Netherlands
Indication	ACS with or without ST elevation	ACS with or without ST elevation, PCI	ACS with or without ST elevation, PCI	STEMI, fibrinolysis	ACS without ST elevation, age ≥70 y
Experimental group	Ticagrelor/aspirin	Ticagrelor/aspirin	Ticagrelor/aspirin	Ticagrelor/aspirin	Clopidogrel/aspirin
Comparison group	Clopidogrel/aspirin	Clopidogrel/aspirin	Clopidogrel/aspirin	Clopidogrel/aspirin	Ticagrelor/aspirin (5% prasugrel/aspirin)
Duration of follow‐up	12 mo	12 mo	12 mo	30 d	12 mo
Primary end point	Cardiovascular death, MI, stroke: 9.8% vs 11.7%; HR, 0.84 [95% CI, 0.77–0.92; *P*<0.001]	Co‐primary ischemia and bleeding end points	PLATO major or minor bleeding: 11.7% vs 5.3%; HR, 2.26 [95% CI, 1.34–3.79; *P*=0.002]	TIMI major bleeding: 0.73% vs 0.69%; absolute difference 0.04%; 95% CI 0.49–0.58; *P*<0.001 for noninferiority	PLATO major or minor bleeding: 18% vs 24%; HR, 0.71 [95% CI 0.54–0.94; *P*=0.02] Net clinical benefit of all‐cause death, MI, stroke, PLATO major and minor bleeding: 28% vs 32%; HR, 0.82 [95% CI, 0.66–1.03; *P*=0.11]
Ischemic end point	Cardiovascular death: 4.0% vs 5.1%; HR, 0.79 [95% CI, 0.69–0.91; *P*=0.001] MI: 5.8% vs 6.9%; HR, 0.84 [95% CI 0.75–0.95; *P*=0.005] Stroke: 1.5% vs 1.3%; HR, 1.17 [95% CI, 0.91–1.52; *P*=0.22]	Cardiovascular death, MI, or stroke: 9.0% vs 6.3%; HR, 1.47 [95% CI, 0.88–2.44]	Cardiovascular death, MI, or stroke: 9.2% vs 5.8%; HR, 1.62 [95% CI, 0.96–2.74; *P*=0.07]	Cardiovascular death, MI, or stroke: 4.0% vs 4.3%; HR, 0.91 [95% CI, 0.67–1.25; *P*=0.57]	Cardiovascular death, MI, stroke: 11% vs 12%; HR, 0.92 [95% CI, 0.64–1.34; *P*=0.71]
Bleeding end point	PLATO major bleeding: 11.6% vs 11.2%; HR, 1.04 [95% CI, 0.95–1.13; *P*=0.43] Non‐CABG PLATO major bleeding: 4.5% vs 3.8%; HR 1.19; 95% CI 1.02–1.38; *P*=0.03	PLATO major bleeding: 10.3% vs 6.8%; HR, 1.54 [95% CI, 0.94–2.53]	PLATO major or minor bleeding: 11.7% vs 5.3%; HR 2.26; 95% CI 1.34–3.79; *P*=0.002]	TIMI major bleeding: 0.73% vs 0.69%; absolute difference 0.04%; 95% CI, 0.49–0.58; *P*<0.001 for noninferiority	PLATO major or minor bleeding: 18% vs 24%; HR 0.71 [95% CI, 0.54–0.94; *P*=0.02]

Trial Acronym	PRAGUE‐18 (29)	ISAR‐REACT 5 (30)	ATLANTIC (33)	DUBIUS (34)
Total patients	1230	4018	1862	1449
Study design	Open‐label	Open‐label	Double‐blind	Open‐label
Enrollment period	April 2013–May 2016	September 2013–February 2018	September 2011–October 2013	December 2015–May 2020
Publication year	2016	2019	2014	2020
Location	Czech Republic	Germany, Italy	Multinational	Italy
Indication	STEMI, primary PCI	ACS with or without ST elevation, PCI	STEMI, primary PCI	ACS without ST elevation, PCI
Experimental group	Prasugrel	Ticagrelor	Prehospital ticagrelor	No ticagrelor treatment
Comparison group	Ticagrelor	Prasugrel	In‐hospital ticagrelor	Ticagrelor pretreatment
Duration of follow‐up	7 d	1 y	30 d	30 d
Primary end point	Composite of all‐cause death, reinfarction, urgent target vessel revascularization, stroke, bleeding requiring transfusion or prolonged hospitalization: 4.0% vs 4.1%; OR, 0.98 [95% CI, 0.55–1.73; *P*=0.94]	Composite of all‐cause death, MI, stroke: 9.3% vs 6.9%; HR, 1.36 [95% CI, 1.09–1.70; *P*=0.006]	Absence of ST‐segment elevation resolution ≥70% before PCI: 86.8% vs 87.6%; OR, 0.93 [95% CI, 0.69–1.25; *P*=0.63] Absence of TIMI flow grade 3 in infarct‐related artery at initial angiography: 82.6% vs 83.1%; OR, 0.97 [95% CI, 0.75–1.25; *P*=0.82]	Composite of cardiovascular death, MI, stroke, BARC type 3–5 bleeding: 2.9% vs 3.3%; ARR −0.46; 95% CI, 2.87–1.89; *P*=0.50
Ischemic end point	Composite of cardiovascular death, MI, or stroke at 30 d: 2.7% vs 2.5%; OR 1.06, 95% CI 0.53–2.15; *P*=0.86	Death: 4.5% vs 3.7% MI: 4.8% vs 3.0% Stroke: 1.1% vs 1.0%	Composite of death, MI, stroke, urgent revascularization, or definite stent thrombosis at 30 d: 4.5% vs 4.4%; OR, 1.03 [95% CI, 0.66–1.60; *P*=0.91]	Cardiovascular death: 0.4% vs 0.2% MI: 0.9% vs 0.9% Stroke: 0.2 vs 0.1%
Bleeding end point	TIMI major bleeding at 30 d; 0.6% vs 0.7%; OR, 0.86 [95% CI, 0.17–4.27; *P*=0.85]	BARC type 3–5 bleeding: 5.4% vs 4.8%; HR, 1.12 [95% CI, 0.83–1.51; *P*=0.46]	PLATO major bleeding within 48 h: 1.8% vs 1.6%; *P*=0.76	BARC type 3–5 bleeding: 1.6% vs 1.9%; ARR −0.3; 95% CI, 2.24–1.57

ACS indicates acute coronary syndrome; ARR, absolute risk reduction; BARC, Bleeding Academic Research Consortium; CABG, coronary artery bypass graft; HR, hazard ratio; MI, myocardial infarction; OR, odds ratio; PCI, percutaneous coronary intervention; PLATO, Study of Platelet Inhibition and Patient Outcomes; STEMI, ST‐segment elevation myocardial infarction; and TIMI, Thrombolysis in Myocardial Infarction.

Subsequent comparative studies of DAPT in ACS evaluated East Asian patients who were underrepresented in the PLATO trial where only 6% were East Asian patients and none were Japanese patients. This subgroup is important because these patients have a lower risk for ischemic events and a higher risk for bleeding events for any given level of platelet inhibition compared with Western populations.^16^ PHILO enrolled mostly Japanese patients and found that both PLATO major bleeding and the composite of cardiovascular death, MI, and stroke rates were higher with ticagrelor than with clopidogrel, but the trial was underpowered to show statistical differences.^17^ Similarly, TICAKOREA enrolled Korean patients and found that PLATO major bleeding was significantly higher and that major adverse cardiovascular events were insignificantly higher in patients randomized to DAPT with ticagrelor compared with clopidogrel.^18^

The TREAT trial enrolled patients with ST‐segment elevation myocardial infarction (STEMI) treated with fibrinolytic therapy, a population also excluded from the PLATO trial, and found that DAPT with ticagrelor was non‐inferior to DAPT with clopidogrel in major bleeding at 30 days.^19^ An exploratory efficacy analysis of 1‐year events similarly showed no benefit in cardiovascular death, MI, or stroke with ticagrelor compared with clopidogrel.^20^

Elderly patients with ACS were also underrepresented in the PLATO trial, a subgroup where bleeding risk is higher. The Popular AGE trial evaluated DAPT with ticagrelor in patients aged ≥70 years with ACS and found that DAPT with clopidogrel resulted in significantly lower major/minor bleeding rates and similar efficacy.^21^ In an observational report from the SWEDEHEART registry, patients at least 80 years old treated with ticagrelor compared with clopidogrel had a relative reduction in the rates of MI and stroke, but an increased risk for death or readmission for bleeding, underscoring that caution should be used in prescribing more potent antiplatelet agents in elderly patients.^22^

The US Food and Drug Administration approved package insert states that ticagrelor is superior to clopidogrel for at least the first 12 months following ACS based on the PLATO trial, a statement supported by clinical practice guidelines.^6^, ^10^ Compared with patients enrolled in clinical trials, however, patients treated in clinical practice have higher risk for ischemic and bleeding events and more comorbidities. Although an initial observational report^23^ published in 2009 supported the PLATO trial results, several subsequent observational reports from more contemporary population cohorts in the Netherlands,^24^ China,^25^ Canada,^26^ Sweden,^27^ the United States and Korea,^28^ and England^29^ have shown similar efficacy with increased bleeding and dyspnea rates for ticagrelor compared with clopidogrel. These studies have the usual observational trial limitations of residual confounding, inaccurate ascertainment of events, and miscoding, but do not have the limitation of selection bias associated with randomized trials and they reached the same conclusion with different study designs. In the PLATO trial, only 60% of patients underwent PCI, 40% with bare metal stents and 60% with first generation stents. In the current era, ischemic risk including stent thrombosis is lower due to advances in stent technology (biocompatible or no polymers, improved anti‐proliferative drugs, thinner stent struts, better delivery platforms), stent implantation optimization with intracoronary imaging, and advances in guideline‐directed medical therapy for secondary prevention. Therefore, bleeding avoidance strategies associated with DAPT have become increasingly important and have changed the balance between ischemic benefit and bleeding risk that determines net clinical benefit for different antiplatelet strategies. Additionally, compared with clopidogrel, the observational outcome reports may have been influenced by increased rates of ticagrelor discontinuation that have been noted in most studies and explained by patient complaints about increased bleeding and dyspnea side effects, inconvenient twice daily administration, and the increased cost of therapy (clopidogrel and prasugrel are generic drugs).

### Comparison of Ticagrelor With Prasugrel

The PRAGUE‐18 study comparing DAPT with ticagrelor or prasugrel in patients with STEMI treated with primary PCI was prematurely stopped for futility because it found no difference in the primary composite outcome of death, reinfarction, urgent target vessel revascularization, stroke, serious bleeding requiring transfusion, or prolonging hospitalization at 7 days or in any individual end points.^30^

Conversely the ISAR‐REACT 5 trial compared DAPT with ticagrelor or prasugrel in a broader population of ACS patients with planned invasive management and found a significantly lower rate of the composite end point of death, MI, or stroke with prasugrel at 1 year without a difference in bleeding risk.^31^ The reduction in ischemic events with prasugrel was unexpected because the trial hypothesis was ticagrelor superiority based on pharmacodynamic studies and indirect comparisons of prior trials. The study has been criticized for its limitations including different loading dose strategies, poor adherence to therapy, event ascertainment by telephone contact, and fewer than expected end point event rates, especially in the prasugrel group.^32^ Observational studies have reported contradictory results.^33^ A meta‐analysis including mostly observational reports concluded that prasugrel might reduce ischemic events at 30 days compared with ticagrelor, but there was no difference in ischemic benefit or bleeding risk at 1 year.^34^ Despite the conflicting data and no obvious pharmacodynamic reason for ticagrelor and prasugrel to yield different clinical results, the 2023 European Society of Cardiology guidelines for the management of acute coronary syndromes gives a Class IIa recommendation to the statement that prasugrel should be considered in preference to ticagrelor for patients proceeding to PCI.^8^

### Timing of Ticagrelor Initiation

Clinical studies have also evaluated the optimal timing of ticagrelor initiation. ATLANTIC investigated prehospital ticagrelor administration in patients with STEMI, but found no improvement in coronary reperfusion before primary PCI when compared with administration 31 minutes later in the catheterization laboratory.^35^ Ischemic outcomes also did not differ, although stent thrombosis was significantly lower at 24 hours and 30 days in the prehospital group.

The DUBIUS trial compared 2 administration strategies.^36^ First, patients with ACS planned for invasive management were randomized to ticagrelor pretreatment approximately 24 hours before angiography versus no pretreatment. Patients with no pretreatment were then randomized to ticagrelor or prasugrel after PCI. There was no difference in the composite end point of cardiovascular death, MI, stroke, or Bleeding Academic Research Consortium (BARC) type 3 to 5 bleeding at 30 days between pre‐treatment or no pre‐treatment, so the study was stopped for futility. An exploratory analysis in the no pretreatment arm showed no difference between ticagrelor and prasugrel.

In summary, ticagrelor decreased ischemic events in patients with ACS compared with clopidogrel in a treatment era when ischemic risk was higher and bleeding complications were less valued, but the benefit is not as obvious in the current era with lower ischemic risk and where bleeding avoidance strategies and net clinical benefit are emphasized.

## Low‐Moderate Risk Ischemic Stroke and High‐Risk TIA

The risk of a recurrent event in the first few months after ischemic stroke or TIA is 5% to 10%. Aspirin decreases that risk. The SOCRATES trial showed that ticagrelor monotherapy was no better than aspirin monotherapy in preventing recurrent stroke, MI, or death within 90 days in patients with low‐moderate risk acute ischemic stroke or high‐risk TIA (Table 3).^37^ Additionally, there were no differences in PLATO major bleeding or intracerebral hemorrhage rates, although rates of minor bleeding and dyspnea were higher with ticagrelor. However, when DAPT with ticagrelor was compared with aspirin monotherapy in patients with low‐moderate risk acute ischemic stroke or high‐risk TIA who were not undergoing thrombolysis or endovascular therapy in the THALES trial,^38^ there was a significant reduction in the 30‐day primary composite outcome of recurrent stroke or death. The difference was driven by a lower risk of recurrent ischemic stroke with DAPT with no difference in mortality or disability rates. The incidence of GUSTO severe bleeding, intracranial hemorrhage, or fatal bleeding was uncommon, but higher with DAPT. The number needed to treat to prevent one primary outcome event was 92, with a number needed to harm of 263 for a severe bleeding event.

**Table 3:** Low‐Moderate Risk Stroke or High‐Risk TIA RCTs

Trial Acronym	SOCRATES (35)	THALES (36)
Total patients	13 199	11 016
Study design	Double‐blind	Double‐blind Placebo controlled
Enrollment period	January 2014–October 2015	January 2018–October 2019
Publication year	2016	2020
Location	Multinational	Multinational
Indication	Mild/Moderate ischemic stroke or high‐risk TIA	Mild/Moderate ischemic stroke or high‐risk TIA
Experimental group	Ticagrelor	Ticagrelor/aspirin
Comparison group	Aspirin	Aspirin
Duration of follow‐up	90 d	30 d
Primary end point	MI, stroke, all‐cause death: 6.7% vs 7.5%; HR, 0.89 [95% CI, 0.78–1.01; *P*=0.07]	Stroke or all‐cause death: 5.5% vs 6.6%; HR, 0.83 [95% CI, 0.71–0.96; *P*=0.02]
Ischemic end point	Ischemic stroke: 5.8% vs 6.7%; HR, 0.87 [95% CI, 0.76–1.00; *P*=NS]	Ischemic stroke: 5.0% vs 6.3%; HR 0.79 [95% CI, 0.68–0.93; *P*=0.004]
Bleeding end point	PLATO major bleeding: 0.5% vs 0.6%; HR, 0.83 [95% CI, 0.52–1.34; *P*=0.45] Intracranial Hemorrhage: 0.2% vs 0.3%; HR, 0.68 [95% CI, 0.33–1.41; *P*=0.30]	GUSTO major bleeding: 0.5% vs 0.1%; HR, 3.99 [95% CI, 1.74–9.14; *P*=0.001] Intracranial hemorrhage: 0.4% vs 0.1%; HR, 3.33 [95% CI, 1.34–8.28; *P*=0.01]

GUSTO indicates Global Utilization of Streptokinase and t‐PA for Occluded Coronary Arteries; HR, hazard ratio; MI, myocardial infarction; PLATO, Study of Platelet Inhibition and Patient Outcomes; and TIA, transient ischemic attack.

Two trials have similarly shown that DAPT with clopidogrel reduces risk for recurrent ischemic stroke in patients with mild ischemic stroke or high‐risk TIA compared with aspirin monotherapy. In the CHANCE trial, the 90‐day outcome of ischemic or hemorrhagic stroke in Chinese patients was reduced (8.2% versus 11.7%; HR, 0.68 [95% CI, 0.57–0.81; *P*<0.001]) without an increase in bleeding events.^39^ Moreover, the 90‐day composite end point of recurrent ischemic stroke, MI, or cardiovascular death was also significantly lower with DAPT in the POINT trial (5.0% versus 6.5%; HR, 0.75 [95% CI, 0.59–0.95; *P*=0.02]), although major and minor bleeding rates were higher.^40^ The POINT investigators estimated that 15 ischemic events would be prevented and 5 major bleeding events would result from treating 1000 patients with DAPT for 90 days. A recent meta‐analysis confirmed that DAPT compared with aspirin monotherapy reduces the risk of recurrent stroke with a small, but significant, increased risk for major bleeding and no difference in risk for all‐cause mortality.^41^

In the CHANCE‐2 trial, only Chinese patients with minor ischemic stroke or high‐risk TIA who were CYP2C19 loss‐of‐function allele carriers were enrolled.^42^ They were randomized to DAPT with ticagrelor or DAPT with clopidogrel, with aspirin stopped after 21 days. The 90‐day outcome of new stroke was significantly lower with ticagrelor (6.0% versus 7.6%; HR, 0.77 [95% CI, 0.64–0.94; *P*=0.008]), with no difference in major or moderate bleeding, but mild bleeding was increased.

In November 2020, the US Food and Drug Administration granted an indication extension for DAPT with ticagrelor, 90 mg, in patients with mild–moderate acute ischemic stroke or high‐risk TIA for up to 30 days to reduce the risk of recurrent neurologic events. In the 2021 AHA/ASA guideline for the prevention of stroke,^11^ DAPT with clopidogrel for 21 to 90 days is favored for patients with stroke (NIH Stroke Scale ≤3) or high‐risk TIA (ABCD score ≥4) (Class I). DAPT with ticagrelor for 30 days is given a lower recommendation (Class IIb) because of increased serious bleeding risk, including intracerebral hemorrhage.

In summary, in patients with low‐moderate risk stroke and high‐risk TIA, DAPT with clopidogrel for 21 to 90 days has shown benefit over aspirin monotherapy in patients with low bleeding risk when treatment is initiated within 12 to 24 hours. DAPT with ticagrelor for 30 days has the advantage in patients with CYP2C19 loss of function alleles who may not respond to clopidogrel. Most of the benefit with DAPT occurs within the first 7 days of treatment. Other patients should only be treated with aspirin monotherapy, clopidogrel monotherapy, or the combination of aspirin and extended‐release dipyridamole (Class I).

## PERIPHERAL ARTERY DISEASE  

Peripheral artery disease is associated with a high risk for cardiovascular death, MI, and ischemic stroke. EUCLID randomized patients with symptomatic peripheral artery disease to monotherapy with ticagrelor or clopidogrel (Table 4).^43^ There was no difference in the composite outcome of cardiovascular death, MI, or ischemic stroke at 30 months, nor were there differences in acute limb ischemia or TIMI major bleeding. The US Food and Drug Administration has approved cilostazol, clopidogrel, vorapaxor, and rivaroxaban for the treatment of peripheral artery disease, but not ticagrelor.

**Table 4:** Atherosclerotic Vascular Disease RCTs

Trial Acronym	EUCLID (41)	ALPHEUS (42)	DACAB (43)	TiCAB (44)	POPular CABG (45)
Total patients	13 855	1910	500	1859	499
Study design	Double‐blind	Open‐label	Open‐label	Double‐blind Placebo controlled	Double blind Placebo controlled
Enrollment period	December 2012–March 2014	January 2017–May 2020	July 2014–November 2015	April 2013–April 2017	March 2015–January 2019
Publication year	2017	2020	2018	2019	2020
Location	Multinational	France, Czech Republic	China	Germany, Austria, Switzerland	Netherlands
Indication	Symptomatic PAD	Elective high‐risk PCI	Elective CABG	Elective CABG	Elective CABG
Experimental group	Ticagrelor	Ticagrelor	Ticagrelor/aspirin or ticagrelor	Ticagrelor	Ticagrelor/aspirin
Comparison group	Clopidogrel	Clopidogrel	Aspirin	Aspirin	Aspirin
Duration of follow‐up	30 mo	48 h	1 y	1 y	1 y
Primary end point	Composite of cardiovascular death, MI, ischemic stroke: 10.8% vs 10.6%; HR, 1.02 [95% CI, 0.92–1.13; *P*=0.65]	Composite of Type 4 MI or major myocardial injury: 35% vs 36%; OR, 0.97 [95% CI, 0.80–1.17; *P*=0.75]	SVG patency: DAPT 88.7%, Ticagrelor 82.8%, aspirin 76.5% DAPT vs aspirin: difference 12.2%, [95% CI 5.2%–19.2%; *P*<0.001] Ticagrelor vs aspirin: Difference 6.3%, [95% CI, 1.1% vs 13.7%, *P*=0.10]	Composite of cardiovascular death, MI, repeat revascularization, stroke: 9.7% vs 8.2%; HR, 1.19 [95% CI, 0.87–1.62; *P*=0.28]	SVG occlusion: 9.6% vs 10.1%; OR, 0.87 [95% CI, 0.49–1.55; *P*=0.64]
Ischemic end point	Acute limb ischemia: 1.7% vs 1.7%: HR, 1.03 [95% CI, 0.79–1.33; *P*=0.85]	MI: 9% vs 8%; OR, 1.03 [95% CI, 0.63–1.68; *P*=0.90]	Composite of death, MI, stroke: DAPT 1.8%, Ticagrelor 2.4%, aspirin 5.4% *P*=NS	Composite of cardiovascular death, MI, stroke: 6.3% vs 6.5%; HR, 0.99, [95% CI, 0.69–1.42; *P*=0.94]	SVG occlusion, SVG revascularization, MI in SVG territory, or sudden death: 12.9% vs 13.0%; HR, 1.04 [95% CI, 0.63–1.69; *P*=0.89]
Bleeding end point	TIMI major bleeding: 1.6% vs 1.6%; HR, 1.10 [95% CI, 0.84–1.43; *P*=0.49]	BARC type 3 or 5 major bleeding: 1 vs 0; *P*=0.5	TIMI major bleeding: DAPT 1.8%, Ticagrelor 1.2%, aspirin 0% *P*=NS	BARC type 3–5 major bleeding: 3.7% vs 3.2%; HR, 1.17 [95% CI, 0.71–1.92; *P*=0.53]	BARC type 3–5 major bleeding: 2.8% vs 3.2%; HR, 0.87 [95% CI, 0.32–2.40; *P*=0.79]

ACS indicates acute coronary syndrome; BARC, Bleeding Academic Research Consortium; CABG, coronary artery bypass graft; DAPT, dual antiplatelet therapy; HR, hazard ratio; MI, myocardial infarction; OR, odds ratio; PAD, peripheral artery disease; PCI, percutaneous coronary intervention; SVG, saphenous vein graft; and TIMI, Thrombolysis in Myocardial Infarction.

In summary, there is no difference between ticagrelor or aspirin monotherapy in patients with peripheral artery disease.

## CORONARY ARTERY DISEASE

DAPT with clopidogrel is recommended for PCI in patients with chronic CAD. ALPHEUS randomized patients undergoing high‐risk elective PCI to DAPT with either ticagrelor or clopidogrel (Table 4).^44^ There were no differences in the rates of periprocedural MI or myocardial injury (troponin elevated greater than 5 times the upper limit of normal without clinical ischemia) within 48 hours or in BARC type 3 or 5 major bleeding, nor were there differences in ischemic end points at 30 days, but there were higher rates of nuisance or minor bleeding, dyspnea, and treatment discontinuation with ticagrelor.

CABG produces an intense inflammatory reaction and activated platelet function. Aspirin improves saphenous vein graft patency and reduces ischemic events.^6^ Studies of DAPT with clopidogrel after CABG have produced conflicting results.^6^ There have been 3 recent trials evaluating ticagrelor for this indication, also with conflicting results. The DACAB trial randomized patients to DAPT with ticagrelor, ticagrelor monotherapy, or aspirin monotherapy.^45^ Compared with aspirin monotherapy, DAPT significantly improved vein graft patency at 1 year, due to the unexpectedly high 23.5% occlusion rate in the aspirin monotherapy group, whereas ticagrelor monotherapy did not. The TiCAB study randomized patients to ticagrelor monotherapy or aspirin monotherapy.^46^ There was no difference in the composite outcome of cardiovascular death, MI, repeat revascularization, and stroke at 1 year in an interim analysis, or in the individual ischemic end points, so the trial was stopped for futility. The POPular‐CABG trial randomized patients to DAPT with ticagrelor versus aspirin monotherapy with no difference in vein graft occlusion at 1 year.^47^ One meta‐analysis including observational trials and older randomized trials found that DAPT with ticagrelor or clopidogrel improved vein graft patency rates compared with aspirin monotherapy, but increased major bleeding.^48^ Another meta‐analysis of 4 ticagrelor randomized clinical trials also showed that DAPT with ticagrelor compared with aspirin monotherapy decreased vein graft failure rates, but increased clinically important bleeding events with no difference in major cardiovascular and cerebrovascular events.^49^ Additionally, there were no differences between ticagelor monotherapy and aspirin monotherapy.

The 2018 ESC/EACTS myocardial revascularization guidelines states that there is insufficient evidence to generally recommend DAPT to reduce saphenous vein graft failure in patients with chronic CAD.^9^ Conversely, the 2021 ACC/AHA/SCAI guideline for coronary artery revascularization states that in selected patients undergoing CABG, DAPT with ticagrelor or clopidogrel for 1 year may be reasonable to improve vein graft patency compared with aspirin alone (Class IIb).^10^

In summary, there is no benefit with substituting ticagrelor for clopidogrel in elective PCI. DAPT with clopidogrel or ticagrelor may improve saphenous vein bypass graft patency rates, but bleeding risk is increased and there is no reduction in major adverse clinical events.

## SECONDARY PREVENTION

DAPT with ticagrelor for long‐term secondary prevention has been evaluated in 2 trials with similar results, but different conclusions. PEGASUS‐TIMI 54 enrolled patients with a history of MI 1 to 3 years before randomization and at least one additional atherothrombotic risk factor (age ≥65 years, diabetes requiring medication, a second prior spontaneous MI, multivessel CAD, or chronic renal dysfunction) to DAPT with either ticagrelor 90 mg or ticagrelor 60 mg or to aspirin monotherapy (Table 5).^50^ After a median follow‐up of 33 months, DAPT with ticagrelor compared with aspirin monotherapy resulted in a significant 1.19% (90 mg) or 1.27% (60 mg) absolute reduction in the primary composite end point of cardiovascular death, MI, or stroke and a significant 1.54% (90 mg) or 1.24% (60 mg) absolute increase in TIMI major bleeding. The annualized efficacy benefit compared with aspirin monotherapy was 4.0 (90 mg) or 4.2 (60 mg) ischemic events prevented per 1000 patients treated. The annualized safety risk with ticagrelor 90 mg was 4.1 (90 mg) or 3.1 (60 mg) excess TIMI major bleeding events per 1000 patients treated. There was no excess in fatal bleeding or intracranial hemorrhage, but minor bleeding and transfusion rates were higher with DAPT, as were discontinuation rates. The authors concluded that DAPT with ticagrelor compared with aspirin monotherapy was efficacious, a message amplified in multiple subsequent substudy reports.

**Table 5:** Secondary Prevention RCTs

Trial Acronym	PEGASUS‐TIMI 54 (48)	THEMIS (49)
Total patients	21 162	19 220
Study design	Double‐blind Placebo controlled	Double‐blind Placebo controlled
Enrollment period	Oct 2010‐May 2013	Feb 2014‐May 2016
Publication year	2015	2019
Location	Multinational	Multinational
Indication	MI 1 to 3 y earlier	CAD, diabetes, no history of MI or stroke
Experimental group	Ticagrelor 90 mg/aspirin Ticagrelor 60 mg/aspirin	Ticagrelor/aspirin
Comparison group	Aspirin	Aspirin
Duration of follow‐up	33 mo	39.9 mo
Primary end point	Cardiovascular death, MI, stroke: 90 mg: 7.85% vs 9.04%; HR, 0.85 [95% CI, 0.75–0.96; *P*=0.008] 60 mg: 7.77% vs 9.04%; HR, 0.84% [95% CI, 0.74–0.95; *P*=0.004]	Cardiovascular death, MI, stroke: 7.7% vs 8.5%; HR, 0.90 [95% CI, 0.81–0.99; *P*=0.04]
Ischemic end point	MI: 90 mg: 4.40% vs 5.25%; HR 0.81, 95% CI 0.69–0.95; *P*=0.01 60 mg: 4.53% vs 5.25%; HR, 0.84 [95% CI, 0.72–0.98; *P*=0.03]	MI: 2.8% vs 3.4; HR, 0.84 [95% CI, 0.71–0.98]
Bleeding end point	TIMI major bleeding: 90 mg: 2.60% vs 1.06%; HR, 2.69 [95% CI, 1.96–3.70; *P*<0.001] 60 mg: 2.30% vs 1.06%; HR, 2.32 [95% CI, 1.68–3.21; *P*<0.001]	TIMI major bleeding: 2.2% vs 1.0%; HR 2.32 [95% CI, 1.82–2.94] *P*<0.001 Intracranial hemorrhage: 0.7% vs 0.5%; HR, 1.71 [95% CI, 1.18–2.48; *P*=0.005]

CAD indicates coronary artery disease; HR, hazard ratio; MI, myocardial infarction; PCI, percutaneous coronary intervention; and TIMI, Thrombolysis in Myocardial Infarction.

The THEMIS trial studied DAPT with ticagrelor (90 mg initially, subsequently reduced to 60 mg after publication of the PEGASUS‐TIMI 54 trial) in patients with chronic CAD and diabetes, but without prior MI or stroke.^51^ After a median follow‐up of 40 months, DAPT compared with aspirin monotherapy resulted in a 0.8% absolute reduction in the primary composite end point of cardiovascular death, MI, or stroke and a 1.2% absolute increase in TIMI major bleeding. The annualized efficacy benefit compared with aspirin monotherapy was 2.14 ischemic events prevented per 1000 patients treated and the annualized safety risk was 2.73 major bleeding events per 1000 patients treated. The minor bleeding rate was increased with DAPT and there was an excess of intracranial hemorrhage events. The investigators concluded that DAPT with ticagrelor was associated with a lower incidence of ischemic events than aspirin monotherapy at the expense of a higher incidence of major bleeding including intracranial hemorrhage, with no risk–benefit difference in the exploratory outcome of net irreversible harm defined as the composite of death, MI, stroke, fatal bleeding, or intracerebral hemorrhage (10.1% versus 10.8%; HR, 0.93 [95% CI, 0.86–1.02]).

However, a substudy report (THEMIS‐PCI) that included 58% of the patients who underwent prior PCI was published the same year, titled as a randomized trial, and widely promoted as proving the benefit of DAPT despite actually being a prespecified substudy analysis of a neutral trial.^52^ DAPT with ticagrelor in patients with a history of PCI resulted in a significant 1.3% absolute reduction in the primary composite end point of cardiovascular death, MI, or stroke and a significant 0.9% absolute increase in TIMI major bleeding over 40 months compared with aspirin monotherapy. Post hoc net clinical benefit was demonstrated for the composite of all‐cause mortality, STEMI, stroke, fatal bleeding, and intracerebral hemorrhage; but no net clinical benefit was seen for the combined original primary efficacy and safety variables of cardiovascular death, MI, stroke, or TIMI major bleeding. No benefit was seen in the subgroup without PCI. The investigators concluded that DAPT with ticagrelor should be considered in patients with diabetes and a history of PCI who have tolerated antiplatelet therapy, have high ischemic risk, and have low bleeding risk, despite the accepted standard that clinical recommendations should not be based on analyses of post‐randomization subgroups.

The HOST‐EXAM trial found benefit for extended clopidogrel monotherapy over aspirin monotherapy after 6 to 18 month of DAPT for the combined end point of all‐cause death, MI, stroke, readmission for ACS, or BARC type 3 to 5 bleeding; but there were no differences in death, MI, or stent thrombosis rates.^53^, ^54^ The benefit was due to reduced rates of stroke, hospitalization for ACS, and bleeding in an East Asian population where clopidogrel is less well metabolized than in other populations. In contrast, there was no benefit for ticagrelor monotherapy versus aspirin monotherapy in a landmark analysis from the GLOBAL LEADERS trial between 1 and 2 years after randomization.^55^ A recent meta‐analysis of randomized patients receiving either P2Y_12_ inhibitor monotherapy or aspirin monotherapy for secondary prevention concluded that the risk of all‐cause death, vascular death, and stroke did not differ, but that the risk of MI was marginally lower in those receiving a P2Y_12_ inhibitor, with a number needed to treat of 244 patients to prevent one event.^56^ Two network meta‐analyses of P2Y_12_ inhibitor monotherapy or aspirin monotherapy after short‐term DAPT showed similar ischemic and bleeding outcomes.^57^, ^58^ Two additional meta‐analyses also showed no difference between P2Y_12_ inhibitor monotherapy or aspirin monotherapy for secondary prevention.^59^, ^60^ Therefore, although there are theoretical reasons why P2Y_12_ receptor inhibition might have clinical advantages over cyclooxygenase enzyme inhibition for secondary prevention, the hypothesis has yet to be proven.

Nevertheless, in September 2015, the US Food and Drug Administration approved long‐term DAPT with ticagrelor, 60 mg, in patients with a history of MI based on the PEGASUS‐TIMI 54 trial and extended the label in June 2020, based on the THEMIS trial, to reduce the risk of first MI or stroke in high‐risk patients with chronic CAD irrespective of diabetes status.

In summary, in patients receiving antiplatelet therapy for secondary prevention, ischemic events are slightly decreased, and bleeding events are slightly increased with prolonged DAPT with ticagrelor compared with aspirin monotherapy, but there is no net clinical benefit. Nor is there obvious clinical benefit with P2Y_12_ monotherapy versus aspirin monotherapy.

## DAPT DE‐ESCALATION

The concept of DAPT de‐escalation to decrease P2Y_12_ inhibitor potency by switching from ticagrelor or prasugrel to clopidogrel or by lowering the ticagrelor or prasugrel dose is based on the observation that ischemic risk is highest in the first month after PCI and then declines significantly, whereas bleeding risk persists during the subsequent months of treatment. The potential benefits of de‐escalation are fewer bleeding events and the reduced cost burden of generic clopidogrel compared with ticagrelor. De‐escalation can be unguided or guided by platelet function testing or CYP2C19 genotype testing.

The TALOS‐AMI trial included Korean patients undergoing PCI for MI who had uneventfully completed 1 month of DAPT with ticagrelor (Table 6).^61^ Compared with continuing DAPT with ticagrelor for 12 months, unguided de‐escalation to DAPT with clopidogrel for the next 11 months met criteria for both noninferiority and superiority for the primary end point of cardiovascular death, MI, stroke, and BARC type 2, 3, or 5 bleeding. The benefit was primarily driven by a reduction in bleeding, but ischemic events were also numerically lower with the de‐escalation strategy. Previously, the small TOPIC trial suggested that unguided de‐escalating from DAPT with ticagrelor or prasugrel 1 month after PCI for ACS to DAPT with clopidogrel significantly reduced bleeding events without an increase in ischemic events.^62^

**Table 6:** DAPT De‐Escalation and Aspirin Withdrawal RCTs

Trial Acronym	TALOS‐AMI (55)	GLOBAL LEADERS (51)	TWILIGHT (64)	TICO (65)
Total patients	2697	15 968	7119	3056
Study design	Open‐label Non‐inferiority	Open‐label	Double‐blind Placebo controlled	Open label
Enrollment period	Feb 2014‐Dec 2018	Jul 2013‐Nov 2015	Jul 2015‐Dec 2017	Aug 2015‐Oct 2018
Publication year	2021	2018	2019	2020
Location	South Korea	Multinational	Multinational	South Korea
Indications	MI with or without ST elevation, Ticagrelor/aspirin for 1 mo	CAD or ACS with or without ST elevation, PCI	PCI, high‐risk for bleeding or ischemic event, ticagrelor/aspirin for 3 mo	ACS with or without ST elevation, PCI
Experimental group	Clopidogrel/aspirin	Ticagrelor/aspirin for 1 mo, followed by Ticagrelor for 23 mo	Ticagrelor	Ticagrelor/aspirin for 3 mo, ticagrelor for 9 mo
Comparison group	Ticagrelor/aspirin	CAD: Clopidogrel/aspirin for 12 mo, followed by aspirin for 12 mo ACS: ticagrelor/aspirin for 12 mo, followed by aspirin for 12 mo	Ticagrelor/aspirin	Ticagrelor/aspirin
Duration of follow‐up	12 mo	24 mo	12 mo	12 mo
Primary end point	Cardiovascular death, MI, stroke, BARC type 2, 3, 5 bleeding: 4.6% vs 8.2%; HR, 0.55 [95% CI, 0.40–0.76; *P*=0.001]	All‐cause death, Q‐wave MI: 3.81% vs 4.37%; RR 0.87 [95% CI, 0.75–1.01; *P*=0.73]	BARC 2, 3, 5 type bleeding: 4.0% vs 7.1%; HR, 0.56 [95% CI, 0.45–0.68; *P*<0.001]	TIMI major bleeding, all‐cause death, MI, stent thrombosis, stroke, target vessel revascularization; 3.9% vs 5.9%; HR 0.66 [95% CI, 0.48–0.92; *P*=0.01]
Ischemic end point	Cardiovascular death, MI, stroke: 2.1% vs 3.1%; HR, 0.69 [95% CI, 0.42–1.14; *P*=0.15]	MI: 3.11% vs 3.13%; RR, 1.00 [95% CI, 0.84–1.19; *P*=0.98] Definite stent thrombosis: 0.80% vs 0.80%; RR, 1.00 [95% CI, 0.71–1.42, *P*=0.98]	All‐cause death, MI, stroke: 3.9% vs 3.9%; HR, 0.99 [95% CI, 0.78–1.25]	All‐cause death, MI, stent thrombosis, stroke, target vessel revascularization: 2.3% vs 3.4%; HR, 0.69 [95% CI, 0.45–1.06; *P*=0.09]
Bleeding end point	BARC type 2,3,5 bleeding: 3.0% vs 5.6%; HR, 0.52 [95% CI, 0.35–0.77; *P*=0.0012]	BARC type 3 or 5 bleeding: 2.04% vs 2.12%, RR, 0.97 [95% CI, 0.78–1.20; *P*=0.77]	BARC 2, 3, 5 type bleeding: 4.0% vs 7.1%; HR, 0.56 [95% CI, 0.45–0.68; *P*<0.001]	TIMI major bleeding: 1.7% vs 3.0%; HR, 0.56 [95% CI, 0.34–0.91; *P*=0.02]

ACS indicates acute coronary syndrome; BARC, Bleeding Academic Research Consortium; CAD, coronary artery disease; HR, hazard ratio; MI, myocardial infarction; OR, odds ratio; PCI, percutaneous coronary intervention; RR, risk reduction; and TIMI, Thrombolysis in Myocardial Infarction.

The TROPICAL‐ACS study reported that DAPT de‐escalation guided by platelet function testing from prasugrel to clopidogrel in clopidogrel responders 2 weeks after PCI was non‐inferior to standard treatment with prasugrel over 12 months, with no increase in ischemic events.^63^

Whereas platelet function testing reports are limited by the use different measuring devices in different populations and varying results over time, genotyping offers a more reproducible result. The PHARMCLO trial randomized patients with ACS to DAPT regimens based on their clinical and genetic characteristics or a standard treatment approach using clinical characteristics alone.^64^ The genomic approach significantly reduced the one‐year composite outcome of cardiovascular death, MI, stroke, or BARC type 3 to 5 major bleeding (15.9% versus 25.9%), a surprising benefit since there was only a 7.4% absolute difference in clopidogrel utilization between the treatment strategies. Two other randomized trials failed to show benefit with the genotype‐guided strategy. The Popular Genetics trial randomized STEMI patients undergoing primary PCI.^65^ In the genotype‐guided strategy, patients with loss‐of‐function alleles were treated with ticagrelor or prasugrel and noncarriers were treated with clopidogrel and compared with patients receiving standard treatment with ticlopidine or prasugrel. There were no differences in the combined outcome of ischemic events or PLATO major bleeding at 1 year, but PLATO minor bleeding was significantly reduced with the genotype‐guided strategy. TAILOR‐PCI randomized patients with ACS or chronic CAD undergoing PCI.^66^ Patients in the genotype‐guided strategy with CYP2C19 loss‐of‐function alleles were treated with ticagrelor and noncarriers were treated with clopidogrel and compared with patients receiving routine clopidogrel therapy. The genotype‐guided approach did not reduce the composite ischemic outcome of cardiovascular death, MI, stroke, stent thrombosis, or severe recurrent ischemia at 12 months or major or minor bleeding rates in the randomized groups or in the subset of patients with CYP2C19 loss of function alleles.

The optimal duration of DAPT with ticagrelor or prasugrel from 1 to 3 months before de‐escalation is still being debated. DAPT de‐escalation is a more attractive strategy in patients without major ischemic or bleeding events after uncomplicated PCI who have lower ischemic risk and higher bleeding risk. It should be noted that most of the benefit in these trials was due to a reduction in minor bleeding, not major bleeding, but importantly, there was no signal for increased risk for ischemic events.

A less popular strategy to decrease bleeding risk is prasugrel dose de‐escalation from 10 to 5 mg instead of substituting for clopidogrel. In the HOST‐REDUCE‐POLYTECH‐ACS trial, this strategy in Korean patients 1 month after ACS was superior to continuing with prasugrel 10 mg daily for 1 year due to a significant reduction in bleeding events without an increase in ischemic events.^67^ There are no published trials of early ticagrelor de‐escalation from 90 mg to 60 mg. A recent network meta‐analysis including 15 randomized trials and 55 798 patients confirmed that the DAPT de‐escalation strategy to clopidogrel or low‐dose prasugrel decreases bleeding events without increasing ischemic events.^68^ Another network meta‐analysis showed better outcomes with the unguided therapy strategy versus the guided therapy strategy.^69^ Support for the guided therapy strategy is further limited by the lack of widespread clinical availability of platelet function or genotype testing and proof of cost effectiveness compared with a more practical and feasible uniform unguided strategy. Moreover, most ischemic events are due to other factors than on‐treatment platelet aggregation results or the presence of loss‐of‐function alleles.

The 2003 European Society of Cardiology guidelines on acute coronary syndromes gives DAPT de‐escalation a Class 2b recommendation as an alternative DAPT strategy.^8^ It states that de‐escalation may be done unguided based on clinical judgment or guided by platelet function testing or CYP2C19 genotyping, depending on the patient's risk profile and the availability of respective assays.

In summary, there is a reduction in bleeding complications with DAPT de‐escalation after 1 to 3 months of DAPT with no increased risk for ischemic complications, an attractive strategy for patients with higher ischemic risk where longer duration DAPT might be preferred.

## Aspirin Withdrawal

Aspirin withdrawal after 1 to 3 months of DAPT is another strategy to reduce bleeding risk. The GLOBAL LEADERS trial tested this strategy in patients with chronic CAD or ACS undergoing PCI, randomizing patients to 1 month of DAPT with ticagrelor followed by 23 months of ticagrelor monotherapy or 12‐months of DAPT (ticagrelor for ACS, clopidogrel for CAD) followed by 12 months of aspirin monotherapy (Table 6).^55^ The aspirin withdrawal strategy did not improve the 2‐year composite primary outcome of all‐cause mortality and Q‐wave MI or the secondary outcome of BARC type 3 or 5 bleeding. In a non‐prespecified post hoc analysis that included 7487 patients with ACS, aspirin withdrawal at 1 month followed by ticagrelor monotherapy decreased 12‐month bleeding compared with DAPT for 12 months (HR, 0.8% versus 1.5% [95% CI, 0.33–0.81; *P*=0.004]), with no difference in all‐cause mortality and Q‐wave MI (1.5% versus 2.0%; HR, 0.73 [95% CI, 0.51–1.03; *P*=0.07]).^70^

In the TWILIGHT trial, patients undergoing PCI were required to have 1 clinical and 1 angiographic feature associated with a high risk of an ischemic or bleeding event.^71^ Aspirin withdrawal after 3 months of uncomplicated DAPT with ticagrelor was compared with continuing DAPT for 12 months. Aspirin withdrawal reduced the primary outcome of BARC type 2, 3, or 5 bleeding events without increasing the composite end point of all‐cause death, nonfatal MI, or stroke. Twenty‐six percent of the bleeding events were BARC type 3 or 5 and they were significantly reduced with aspirin withdrawal (1% versus 2%). These results were maintained among subgroups of patients with non‐ST elevation ACS, diabetes, male or female sex, complex PCI, chronic kidney disease, and high bleeding risk. TWILIGHT was noted to differ from GLOBAL LEADERS in several aspects, including the double‐blind design, randomization after 3 months of DAPT, the high‐risk population, the shorter duration of follow‐up, and the use of adjudicated (versus site reported) bleeding events.

The TICO trial similarly studied 3‐months of DAPT with ticagrelor followed by aspirin withdrawal or continued DAPT with ticagrelor up to 12 months in patients with ACS undergoing PCI.^72^ The trial differed from TWILIGHT in that patients were randomized after PCI rather than at 3 months, patients with STEMI were included, high bleeding risk patients were excluded, and the trial was conducted in South Korea. Aspirin withdrawal significantly reduced net adverse clinical events, the composite of TIMI major bleeding and cardiovascular and cerebrovascular events, as well as TIMI major bleeding events. There were no differences in the individual or combined ischemic end points.

Similar benefit in East Asian patients undergoing PCI was shown for aspirin withdrawal compared with DAPT with clopidogrel after 3 months of DAPT in the SMART‐CHOICE trial^73^ and for aspirin withdrawal after 1 month of DAPT with clopidogrel in the STOPDAPT‐2 trial.^74^ However, in the STOPDAPT‐2 ACS trial, clopidogrel monotherapy after 1 month of DAPT resulted in a higher risk for ischemic events (primarily MI) after PCI for ACS compared with 12 months of DAPT, suggesting that very early withdrawal of aspirin in patients at high ischemic risk may be an inferior strategy.^75^ Conversely, 1 month of DAPT was noninferior to 3 or more months of DAPT after PCI for ischemic events, but decreased bleeding events in patients with higher bleeding risk and lower ischemic risk in the MASTER‐DAPT trial.^76^

As reviewed above, there is no convincing evidence of a therapeutic difference between P2Y_12_ monotherapy and aspirin monotherapy for secondary prevention.^56^, ^57^, ^58^, ^59^, ^60^ In the 2021 ACC/AHA/SCAI revascularization guidelines, 1 to 3 months of DAPT in selected patients undergoing PCI followed by aspirin withdrawal to reduce the risk of bleeding events was given a Class 2a recommendation.^10^

In summary, there is a reduction in bleeding complications with aspirin (or P2Y_12_ inhibitor) withdrawal after 1 to 3 months of DAPT with no increased risk for ischemic complications, an attractive strategy for patients with higher bleeding risk where shorter duration DAPT might be preferred.

## CONCLUSIONS

It is challenging to compare the results of antiplatelet therapy across different randomized clinical trials because of differences in end point definitions, primary end points, and patient populations.^77^ Nevertheless, a review of the ticagrelor randomized clinical trial evidence base, in the context of decreased ischemic risk in the current treatment era and increased emphasis on bleeding risk and net clinical benefit, suggests that clinical conventional wisdom and clinical trial guideline support for the superior benefit of more potent platelet P2Y_12_ receptor inhibition with ticagrelor compared with clopidogrel may be overemphasized.

## Disclosures

None.
